# Multispecies Transcriptomics Data Set of Brugia malayi, Its *Wolbachia* Endosymbiont *w*Bm, and Aedes aegypti across the B. malayi Life Cycle

**DOI:** 10.1128/MRA.01306-18

**Published:** 2018-11-08

**Authors:** Matthew Chung, Laura Teigen, Silvia Libro, Robin E. Bromley, Nikhil Kumar, Lisa Sadzewicz, Luke J. Tallon, Jeremy M. Foster, Michelle L. Michalski, Julie C. Dunning Hotopp

**Affiliations:** a Institute for Genome Sciences, University of Maryland School of Medicine, Baltimore, Maryland, USA; b Department of Microbiology and Immunology, University of Maryland School of Medicine, Baltimore, Maryland, USA; c Department of Biology, University of Wisconsin Oshkosh, Oshkosh, Wisconsin, USA; d New England Biolabs, Ipswich, Massachusetts, USA; e Greenebaum Cancer Center, University of Maryland, Baltimore, Maryland, USA; Indiana University Bloomington

## Abstract

Here, we present a comprehensive transcriptomics data set of Brugia malayi, its Wolbachia endosymbiont *w*Bm, and its vector host. This study samples from 16 stages across the entire B. malayi life cycle, including stage 1 through 4 larvae, adult males and females, embryos, immature microfilariae, and mature microfilariae.

## ANNOUNCEMENT

Brugia malayi is the laboratory model for lymphatic filariasis, one of the most prevalent vector-borne parasitic diseases (1) with more than 856 million individuals at risk in regions of endemicity (2). Here, we present transcriptomic data for B. malayi, its Wolbachia endosymbiont *w*Bm, and the vector host across the entire B. malayi life cycle.

B. malayi were isolated using National Institute of Allergy and Infection Diseases/National Institutes of Health (NIAID/NIH) Filariasis Research Reagent Resource Center (FR3) protocols (www.filariasiscenter.org); all animal care and use protocols were approved by the University of Wisconsin Oshkosh Institutional Animal Care and Use Committee (UWO IACUC). All samples were flash frozen in liquid nitrogen and stored at −80°C before RNA isolation. Aedes aegypti black-eyed, Liverpool-strain mosquitoes were fed on FR3-strain microfilaremic cat blood. Infective third-stage B. malayi larvae (L3) were isolated from whole mosquitoes in bulk and used for infections. Thoraces of infected mosquitoes were collected 18 h postinfection (hpi), 4 days postinfection (dpi), and 8 dpi (Table 1). Male gerbils (Meriones unguiculatus) 3 months old or older from Charles River Laboratories were infected with bulk-purified L3s injected into the peritoneal cavity with sample collection at the required times by terminal worm recovery (Table 1). Eggs and embryos were obtained by cutting adult females and removing uterine tissue using forceps (3).

**TABLE 1:** RNA-Seq samples and SRA accession numbers across the *B. malayi* life cycle

Sample	BioSample accession no.	Enrichment method	SRA accession no.	No. of total sequenced reads
Jird, 1 dpi	SAMN04313629	Poly(A) enrichment	SRX2409229	11,702,630
	SAMN04313629	Poly(A) enrichment	SRX2409230	39,429,554
	SAMN04313633	Poly(A) enrichment	SRX2415911	10,512,230
	SAMN04313633	Poly(A) enrichment	SRX2415910	45,513,518
	SAMN04314682	RiboZero treated, poly(A) depletion	SRX2415979	17,479,084
	SAMN04314682	RiboZero treated, poly(A) depletion	SRX2415978	30,356,424
	SAMN04314686	RiboZero treated, poly(A) depletion	SRX2416090	14,570,410
	SAMN04314686	RiboZero treated, poly(A) depletion	SRX2416089	60,046,756
Jird, 2 dpi	SAMN04313635	Poly(A) enrichment	SRX2414981	12,861,524
	SAMN04313635	Poly(A) enrichment	SRX2414982	42,369,518
	SAMN04313631	Poly(A) enrichment	SRX2415908	13,638,502
	SAMN04313631	Poly(A) enrichment	SRX2415909	47,714,364
	SAMN04314688	RiboZero treated, poly(A) depletion	SRX2416010	11,695,030
	SAMN04314688	RiboZero treated, poly(A) depletion	SRX2416009	44,550,904
	SAMN04314684	RiboZero treated, poly(A) depletion	SRX2416087	2,502,950
	SAMN04314684	RiboZero treated, poly(A) depletion	SRX2416088	53,819,452
Jird, 3 dpi	SAMN04313619	Poly(A) enrichment	SRX1539755	85,614,594
	SAMN04313620	Poly(A) enrichment	SRX1539758	97,373,488
	SAMN04314672	RiboZero treated, poly(A) depletion	SRX1539741	31,671,524
	SAMN04314673	RiboZero treated, poly(A) depletion	SRX1539742	36,061,876
Jird, 4 dpi	SAMN04313621	Poly(A) enrichment	SRX1539799	87,831,322
	SAMN04313622	Poly(A) enrichment	SRX1539813	77,919,818
	SAMN04314674	RiboZero treated, poly(A) depletion	SRX1539745	18,001,022
	SAMN04314674	RiboZero treated, poly(A) depletion	SRX1539744	18,059,474
	SAMN04314675	RiboZero treated, poly(A) depletion	SRX1539747	26,818,742
Jird, 8 dpi	SAMN04313623	Poly(A) enrichment	SRX1539817	84,250,470
	SAMN04313624	Poly(A) enrichment	SRX1539819	84,231,566
	SAMN04314676	RiboZero treated, poly(A) depletion	SRX1539748	41,578,604
	SAMN04314677	RiboZero treated, poly(A) depletion	SRX1539750	33,505,904
Jird, 20 dpi, immature male	SAMN04313625	Poly(A) enrichment	SRX1539862	86,565,686
	SAMN04313626	Poly(A) enrichment	SRX1539865	93,010,092
	SAMN04314678	RiboZero treated, poly(A) depletion	SRX1539752	58,212,484
	SAMN04314679	RiboZero treated, poly(A) depletion	SRX1539754	53,899,664
Jird, 24 dpi, immature female	SAMN04313627	Poly(A) enrichment	SRX1539869	80,399,030
	SAMN04313628	Poly(A) enrichment	SRX1539871	94,819,216
	SAMN04314680	RiboZero treated, poly(A) depletion	SRX1539757	73,230,576
	SAMN04314680	Total RNA, *w*Bm Agilent SureSelect	SRX2508257	124,210,676
	SAMN04314681	RiboZero treated, poly(A) depletion	SRX1539735	77,618,810
Jird, adult male	SAMN04313616	Poly(A) enrichment	SRX1539740	110,125,724
	SAMN04313615	Poly(A) enrichment	SRX1539737	95,537,492
	SAMN04314669	RiboZero treated, poly(A) depletion	SRX1539735	77,618,810
	SAMN04314668	RiboZero treated, poly(A) depletion	SRX1539732	43,412,692
Jird, adult female	SAMN04313613	Poly(A) enrichment	SRX1539730	85,725,576
	SAMN04313614	Poly(A) enrichment	SRX1539734	90,360,386
	SAMN04313611	Poly(A) enrichment	SRX1539085	87,158,676
	SAMN04313612	Poly(A) enrichment	SRX1539707	89,298,942
	SAMN04314666	RiboZero treated, poly(A) depletion	SRX1539729	28,901,790
	SAMN04314667	RiboZero treated, poly(A) depletion	SRX1539731	31,173,032
	SAMN04314667	Total RNA, *w*Bm Agilent SureSelect	SRX2508255	147,440,774
	SAMN04314664	RiboZero treated, poly(A) depletion	SRX1539589	52,924,416
	SAMN04314664	Total RNA, *w*Bm Agilent SureSelect	SRX2508256	124,173,684
	SAMN04314665	RiboZero treated, poly(A) depletion	SRX1539727	54,369,010
Jird, embryo	SAMN04313617	Poly(A) enrichment	SRX1539746	104,139,194
	SAMN04313618	Poly(A) enrichment	SRX1539751	103,938,004
	SAMN04314670	RiboZero treated, poly(A) depletion	SRX1539736	38,620,328
	SAMN04314671	RiboZero treated, poly(A) depletion	SRX1539739	43,744,776
Jird, immature microfilariae	SAMN04313630	Poly(A) enrichment	SRX2415043	12,583,442
	SAMN04313630	Poly(A) enrichment	SRX2415044	35,963,192
	SAMN04313632	Poly(A) enrichment	SRX1539873	54,824,638
	SAMN04314683	RiboZero treated, poly(A) depletion	SRX2416083	17,304,704
	SAMN04314683	RiboZero treated, poly(A) depletion	SRX2416084	32,063,516
	SAMN04314685	RiboZero treated, poly(A) depletion	SRX1539795	42,838,356
Jird, mature microfilariae	SAMN04313636	Poly(A) enrichment	SRX2416293	10,364,900
	SAMN04313636	Poly(A) enrichment	SRX2416292	30,366,668
	SAMN04313636	Poly(A) enrichment	SRX2416292	40,464,760
	SAMN04313634	Poly(A) enrichment	SRX1539875	20,074,600
	SAMN04313634	Poly(A) enrichment	SRX1539874	9,974,198
	SAMN04314689	RiboZero treated, poly(A) depletion	SRX2416086	17,610,260
	SAMN04314689	RiboZero treated, poly(A) depletion	SRX2416085	31,206,204
	SAMN04314687	RiboZero treated, poly(A) depletion	SRX1539797	18,534,882
	SAMN04314687	RiboZero treated, poly(A) depletion	SRX1539798	8,452,542
Vector, 18 hpi	SAMN04313637	Poly(A) enrichment	SRX1539876	103,949,620
	SAMN04313637	Poly(A) enriched, B. malayi Agilent SureSelect	SRX2505171	31,646,034
	SAMN04313637	Poly(A) enriched, B. malayi Agilent SureSelect	SRX2505171	59,222,150
	SAMN04313637	Total RNA, B. malayi Agilent SureSelect	SRX2505170	149,907,628
	SAMN04313638	Poly(A) enrichment	SRX1539886	100,358,314
	SAMN04313638	Poly(A) enriched, B. malayi Agilent SureSelect	SRX2505769	34,175,968
	SAMN04313638	Poly(A) enriched, B. malayi Agilent SureSelect	SRX2505769	63,720,320
	SAMN04314690	RiboZero treated, poly(A) depletion	SRX1539809	30,623,652
	SAMN04314690	Total RNA, *w*Bm Agilent SureSelect	SRX2508248	76,297,602
	SAMN04314691	RiboZero treated, poly(A) depletion	SRX1539810	30,676,768
	SAMN04314691	Total RNA, *w*Bm Agilent SureSelect	SRX2508249	78,959,932
Vector, 4 dpi	SAMN04313639	Poly(A) enrichment	SRX1539947	117,570,910
	SAMN04313639	Poly(A) enriched, B. malayi Agilent SureSelect	SRX2505770	57,037,174
	SAMN04313639	Poly(A) enriched, B. malayi Agilent SureSelect	SRX2505770	106,815,788
	SAMN04313640	Poly(A) enrichment	SRX1539949	113,590,496
	SAMN04313640	Poly(A) enriched, B. malayi Agilent SureSelect	SRX2505771	48,173,212
	SAMN04313640	Poly(A) enriched, B. malayi Agilent SureSelect	SRX2505771	90,429,442
	SAMN04314692	RiboZero treated, poly(A) depletion	SRX1539811	38,453,926
	SAMN04314692	Total RNA, *w*Bm Agilent SureSelect	SRX2508250	64,398,742
	SAMN04314693	RiboZero treated, poly(A) depletion	SRX1539814	30,669,394
	SAMN04314693	Total RNA, *w*Bm Agilent SureSelect	SRX2508252	30,161,844
	SAMN04314693	Total RNA, *w*Bm Agilent SureSelect	SRX2508251	21,071,958
Vector, 8 dpi	SAMN04313641	Poly(A) enrichment	SRX1539952	131,311,710
	SAMN04313641	Poly(A) enriched, B. malayi Agilent SureSelect	SRX2505955	87,079,372
	SAMN04313641	Poly(A) enriched, B. malayi Agilent SureSelect	SRX2505955	164,545,760
	SAMN04313641	Total RNA, B. malayi Agilent SureSelect	SRX2505954	255,771,318
	SAMN04313642	Poly(A) enrichment	SRX1539954	146,982,974
	SAMN04313642	Poly(A) enriched, B. malayi Agilent SureSelect	SRX2505953	108,705,568
	SAMN04313642	Poly(A) enriched, B. malayi Agilent SureSelect	SRX2505953	204,486,026
	SAMN04314694	RiboZero treated, poly(A) depletion	SRX1539815	28,267,988
	SAMN04314694	Total RNA, *w*Bm Agilent SureSelect	SRX2508253	74,052,092
	SAMN04314695	RiboZero treated, poly(A) depletion	SRX1539816	41,159,150
	SAMN04314695	Total RNA, *w*Bm Agilent SureSelect	SRX2508254	70,535,594
Vector, infective L3	SAMN10039708	Poly(A) enriched, B. malayi Agilent SureSelect	SRX4676609	171,398,760
	SAMN10039709	Poly(A) enriched, B. malayi Agilent SureSelect	SRX4676610	171,924,470

For RNA isolations, a 3:1 volume of TRIzol was added to mammalian-stage samples, and 1 ml of TRIzol was added per 50 to 100 mg mosquito tissue. We added β-mercaptoethanol (1%), and the tissues were homogenized using a bead beater and a TissueLyser at 50 Hz for 5 min and then centrifuged at 12,000 × *g* in a fresh tube for 10 min at 4°C. After incubation at room temperature for 5 min, 0.2 volumes of chloroform were added. The samples were shaken by hand for 15 s, incubated at room temperature for 3 min, loaded into a prespun Phase Lock Gel heavy tube, and centrifuged at 12,000 × *g* for 5 min at 4°C. The upper phase was extracted, 1 volume of 100% ethanol was added, and the sample was purified on a PureLink RNA minicolumn following the manufacturer’s instructions. Purified RNA was run on a bioanalyzer. Microfilariae RNA routinely gives atypical profiles and RNA integrity numbers (RINs). Thus, after test sequencing and analysis, all samples were sequenced regardless of the RIN.

Whole-transcriptome libraries for all samples were constructed using the NEBNext Ultra directional RNA library prep kit. For eukaryotic mRNA, the NEBNext poly(A) mRNA magnetic isolation module was used. For prokaryotic mRNA, rRNA and poly(A) reductions were performed as previously described (4, 5). SPRIselect reagent was used for cDNA purification and size selection, a 7-nucleotide index was added by PCR amplification, and 100-bp paired-end reads were generated with an Illumina HiSeq 2500 instrument. B. malayi and *w*Bm Agilent SureSelect custom bait libraries were designed and used, when necessary, as previously described (6), on poly(A)-selected and total RNA libraries, respectively.

These transcriptomics data form a rich data set that will be of immense value to the filarial nematode and Wolbachia research communities for analyzing gene expression as well as structural annotation of nematode and endosymbiont transcripts.

### Data availability.

The data sets supporting the results of this article are available in the Sequence Read Archive (SRA) repository (Table 1). The A. aegypti and B. malayi sequencing reads are available under accession number SRP068692, and the *w*Bm sequencing reads are available under accession number SRP068711.
